# Salivary alpha-amylase: A marker of stress in gynecological residents during a shoulder dystocia simulation scenario

**DOI:** 10.1371/journal.pone.0314234

**Published:** 2024-11-25

**Authors:** Ada Aita, Paola Galozzi, Filippo Zemin, Giulia Principi, Nicole Contran, Giulia Musso, Chiara Cosma, Antonio Ragusa, Donato D’Antona, Daniela Basso

**Affiliations:** 1 Laboratory Medicine Unit, Department of Medicine-DIMED, University of Padova, Padova, Italy; 2 Department of Women and Children’s Health, University of Padova, Padova, Italy; 3 Department of adult and developmental human pathology "G.Barresi", University of Messina, Messina, Italy; 4 Department of Obstetrics and Gynecology, Campus Bio-Medico University Hospital Foundation Rome, Rome, Italy; University of Pisa, ITALY

## Abstract

**Objective:**

Salivary alpha-amylase (sAA) has been recently proposed as biomarker of stress responsiveness within the sympathetic nervous system, preferable to cortisol, since limitations related to cortisol measurement (e.g. diurnal and seasonal rhythms, drugs interferences). Several factors, as age, collection device and analytical methods, also influence sAA levels and interpretation. This study aims to assess whether sAA could be useful to evaluate the stress response, also verifying some sources of variability.

**Methods:**

To identify any sources of sAA variability, saliva samples were collected from eight healthy subjects at five different times (8:00, 10:00, 12:00, 14:00 and 16:00) over five consecutive days using two different collection devices. Saliva was then collected from 35 obstetric residents before and after a simulated shoulder dystocia scenario, one of the most traumatic birth events in the professional life of gynecologists, to assess the stress levels. Samples were analysed throughout two different assays. Heart rate (HR) of residents was also measured before and after simulation scenario. Residents self-collected two saliva samples 10 min apart on a typical day.

**Results:**

Overall, sAA levels increased significantly from morning to afternoon. Levels varied between subjects, but no differences were found between days or sampling devices. sAA activity levels in samples from 35 residents were significantly higher than those obtained before the scenario took place (about ten minutes later). These results were confirmed by two different assays. Moreover, a similar trend was observed when HR was considered. No differences were observed between samples collected 10 minutes apart on a typical day.

**Conclusions:**

Salivary alpha-amylase activity is a reliable, quick, and efficient marker for stress response, then its measurement will be of potential interest in the field of stress-related disorders. However, it is important to consider the timing of sample collection before introducing sAA in a clinical setting.

## Introduction

Saliva caught the attention of the scientific community during the SARS-CoV-2 pandemic as a cost-effective approach to screening and diagnosing infection in large populations [1].

Saliva is an easily accessible body fluid that can be self-collected in a safe and non-invasive way, being of potential value for children and older people, since its collection doesn’t require special equipment or training. Whole saliva contains both locally produced and serum-derived markers, making it a valid alternative to serum for searching biomarkers of diseases and other pathophysiological conditions [2, 3].

Prior to the SARS-CoV-2 pandemic, whole saliva has been widely used as a sample for detecting and measuring drugs, as well as in supporting the diagnosis of endocrinological diseases [4]. More recently, some authors have demonstrated the important role of neuroendocrine markers detectable in saliva in establishing bodily reactions to psychosocial, physical, and biological stress stimuli [5]. Whereas salivary cortisol is a reliable measure for evaluating hypothalamic-pituitary-adrenocortical (HPA) axis activity, salivary alpha amylase (sAA) has been suggested as a marker for sympathetic adrenomedullary (SAM) systems activity [6, 7]. The HPA response, which has been extensively studied for decades, is a hormone cascade ultimately leading to the release of cortisol. It is characterized by a time delay of approximately 20 minutes and longer-lasting effects than the sympathetic response [8]. In contrast, the SAM response appears immediate, as evidenced by the increase in heart rate (HR) and blood pressure as well as pupil dilation [9]. Alpha amylase, secreted by salivary glands, is controlled by autonomic nervous signals. Its increase under various physically (e.g. exercise, heat and cold) and psychologically (e.g. written examinations) stressful conditions in human subjects reflects the activity of the SAM system [10, 11].

In this context, to overcome limitations of cortisol regarding delayed release and measurement bias (e.g. diurnal and seasonal rhythms, drugs interferences), sAA is increasingly being studied as a potential biomarker of stress [12–15].

Alpha amylase is the most abundant salivary enzyme in humans, and a well-known digestive enzyme that breaks down starch. Unlike salivary cortisol, which is produced in endocrine glands and subsequently diffused into saliva, sAA is directly produced by the acinar cells of the salivary glands, mainly by the parotid glands (80% of the total sAA) [16]. The levels of sAA are very low in young infants, who do not have to digest complex carbohydrates yet. Adult levels of sAA are typically reached by the age of 5 or 6, when the diet is similar to that of an adult [17], and they increase across the adult lifespan, older individuals displaying higher overall daily levels when compared to younger ones [18]. A typical diurnal rhythm of sAA activity with a pronounced decrease in the first 30–60 min after awakening, and steadily rising levels towards the afternoon and evening has been demonstrated [19, 20].

Further differences in sAA levels due to age, gender, steroid-related factors, somatic health, acute medication, smoking, consumption of food, drinks and alcohol, physical activity, sleep, collection methods and devices are also documented in the literature [21].

Several methods have been used to collect saliva, including passive drool, Salivette® and eluted filter paper. Each one has different characteristics that can influence analytical results. Moreover, several other commercial collection devices are available [22, 23].

As the use of sAA as a measure of psychological stress in research has increased, several factors should be verified and/or investigated, as intra- and inter-day variations, intra- and inter-individual variability, differences due to collection methods, and methodological issues in measurements of sAA.

In this study, we firstly aimed to verify some sources of variability in sAA activity levels, with the focus on saliva collection devices and analytical methods. Then, we aimed to investigate whether sAA levels could serve as a valid biomarker of stress. To achieve this, we measured sAA levels in the saliva of resident gynecologists during a simulation scenario of shoulder dystocia, which is widely recognized as one of the most traumatic birth events in the professional lives of midwives and gynecologists.

## Materials and methods

### Study design and participants

To verify intra- and inter-individual diurnal variations in sAA activity levels and to evaluate analytical performance characteristics of salivary assays, eight healthy subjects (3 females and 5 males with a mean age ± SD of 32±3.9 years) were enrolled. Each subject self-collected saliva samples at five different time points (at: 8:00, 10:00, 12:00, 14:00 and 16:00) over five consecutive days, from Monday to Friday, using two different devices at each scheduled time.

To assess the role of sAA in stress response, 35 residents (28 females and 7 males with a mean age±SD of 32±2.1 years) attending the third, fourth, and fifth year of the Gynecology and Obstetrics Specialization School of the University of Padova were recruited. As part of their training, residents participated for the first time of their career in the management of an obstetric emergency known as shoulder dystocia, which was reproduced through a simulation scenario. On simulation day, residents self-collected two saliva samples with two different devices at three different times (at 8:00 which was considered the baseline, before simulation, and soon after simulation). To evaluate the stress response to the emergency scenarios and not to the presence of other known people, simulations were conducted in a separate room with trainers and one resident at a time, without the presence of other participants. Simulation scenario lasted approximately 10 minutes and was the same for all residents. To further assess the stress levels of participants, HR was measured to each participant at baseline, immediately before and soon after the traumatic birth scenario.

Moreover, the residents collected two saliva samples after ten minutes each other on an ordinary working day, using the same collection devices and at same time points as the simulation day.

The study has been performed according to the Declaration of Helsinki and it was approved by the Local Ethics Committee (Prot. Nr. 5668/AO/23). Written informed consent was obtained from all individuals included in this study.

### Saliva samples and sAA analysis

Two saliva samples were obtained at each collection time, using first the Salivette® device (SARSTEDT AG & Co, Nümbrecht, Germany) and then the Esaliva device (ESAMED, Vigonza, Italy).

To ensure the quality of the sample collection, the researchers provided a face-to-face demonstration to both healthy subjects and residents on the proper use of Salivette and Esaliva devices.

To minimize any potential physiological effects about responses to sAA, one hour before the beginning of the saliva collection, participants were not allowed eating, exercising, using toothpaste, chewing gum, or drinking.

After collection, saliva samples were sent to the laboratory and centrifuged at 3,500 x g at 4°C for 5 minutes. All samples were then frozen and stored at -20°C for no more than two weeks before performing the analyses.

Saliva samples collected from the eight healthy subjects were analyzed by an enzymatic colorimetric assay for the quantitative determination of α-amylase activity in human serum, plasma and urine (AMYL2) on the Roche Cobas 8000 analyzer (Roche, Basel, Switzerland). Immediately before the analysis, all saliva samples were diluted 1:100 in NaCl 9% diluent, provided by the manufacturer. The analyses were conducted according to the manufacturer’s instructions. Performances characteristics of the method in saliva diluted samples were also verified (S1 File, S1 Fig and S1 Table).

To assess the analytical performances of different assays, all saliva samples collected by residents were analyzed using two different methods: the previously mentioned AMYL2 test (quantitative determination of α-amylase) and a kinetic enzyme assay kit (Salimetrics, State College, PA) specifically designed by the manufacturer to standardize the detection of alpha-amylase in saliva samples. The analyses by using salimetrics kit were conducted according to the manufacturer’s instructions.

### Heart rate (HR) measurement

A professional digital pulse oximeter via multiparametric monitor was used to measure HR.

### Statistical analysis

All statistical analyses were performed using GraphPad Prism software version 6.0 (GraphPad Software Inc).

After confirming that sAA values were not normally distributed using the Shapiro-Wilk test, non-parametric methods were used. The sAA values measured for five days, at the five collection time points, in eight subjects were compared using the Friedman test (F), and Dunn’s multiple comparisons. The sAA levels measured in males and females at the baseline (8.00 a.m.) were compared using Mann-Withney test (MW). The Wilcoxon signed-rank test (W) was then performed for post-hoc and pairwise comparisons. Spearman’s correlation and Bland-Altman plot were used to compare the values of sAA activity obtained from the two different commercial assays.

Repeated measures ANOVA, t-test for paired data were used to compare HR and sAA values measured at baseline, before and after simulation.

## Results

### Diurnal and inter-individual variations in sAA activity levels

**Fig 1:**
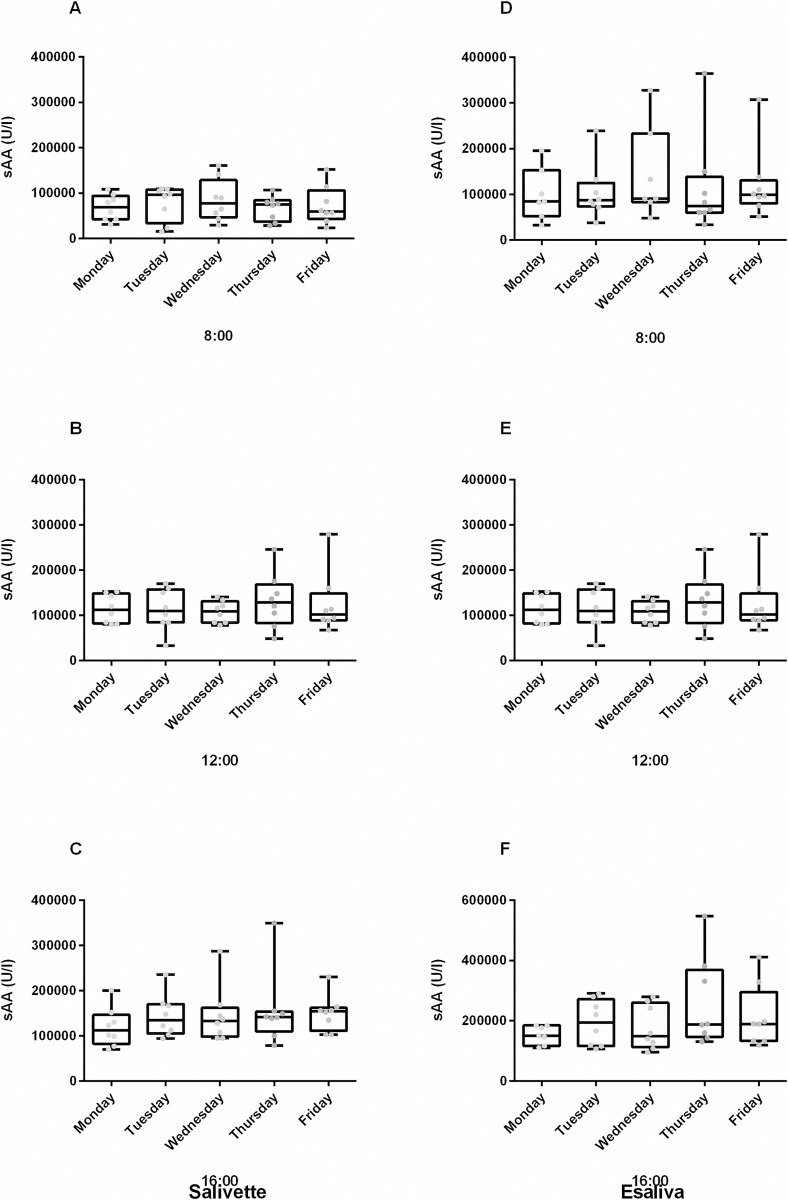
Inter-day sAA levels at three fixed time points. The Figures show the box diagram of the pattern followed by sAA activity (U/L) of the eight enrolled subjects through the five days, at three fixed time points (at 8.00, 12.00 and 14.00) when samples were collected by Salivette (A-C) or Esaliva (D-F). The box plots show median, upper and lower quartiles, minimum and maximum data values. The grey dots represent data distribution.

Day-to-day analysis of sAA activity at fixed time points showed no significant differences among different days, regardless the collection devices. Representative data of three time points (8.00, 12.00 and 16.00) being shown in Fig 1. The analysis of the individual activity of sAA at 8.00 o’ clock demonstrated a significant inter-individual variation (F = 19.93, p = 0.0057, for Salivette and F = 16.98, p = 0.0017, for Esaliva), regardless of the used collection devices. Furthermore, no significant differences in sAA activity were observed between the males (n = 12) and females (n = 31), when sAA baseline levels of the 8 healthy subjects and the 35 residents were evaluated for both Salivette and Esaliva (MW = 120, p = 0.559 and MW = 78, p = 0.0883, respectively).

**Fig 2:**
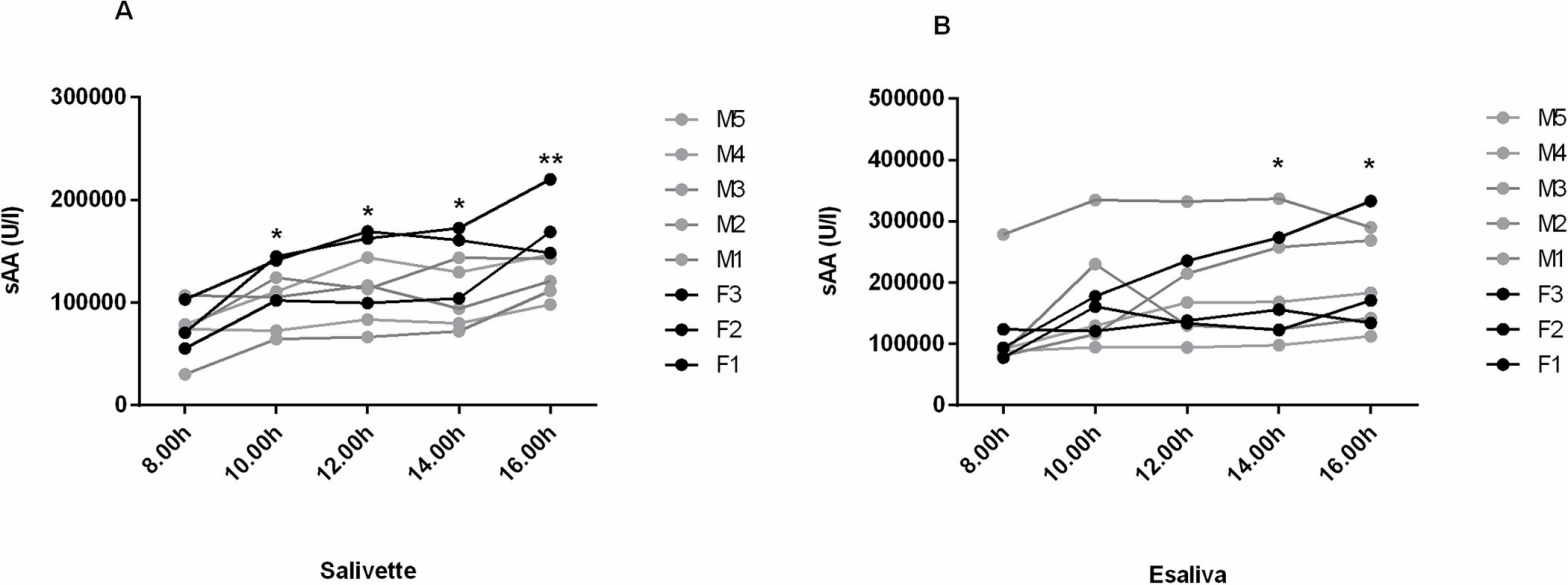
Diurnal sAA levels in eight subjects. Figures show mean levels of sAA activity obtained for each subject at different collection time during five days on saliva samples collected by Salivette (A) and Esaliva (B), respectively. Males and females were indicated by the M and F letters, respectively. Dunn’s multiple comparisons test: * = p<0.05 with respect to 8.00; ** = p<0.05 with respect to 8.00 and 10.00.

A significant increase from morning to the afternoon (F = 13.77, p = 0.0008, for Salivette and F = 20.60, p = 0.0004, for Esaliva) was observed when activity levels of sAA, obtained at the five scheduled collection times during the five days, were analyzed (Fig 2).

### Comparison of the basal sAA levels in samples collected by 35 residents with the two different devices: Salivette and Esaliva

Before assessing sAA as a stress biomarker, differences in sAA levels collected by 35 residents using the two different devices at the baseline (8.00 a.m.) were investigated.

**Fig 3:**
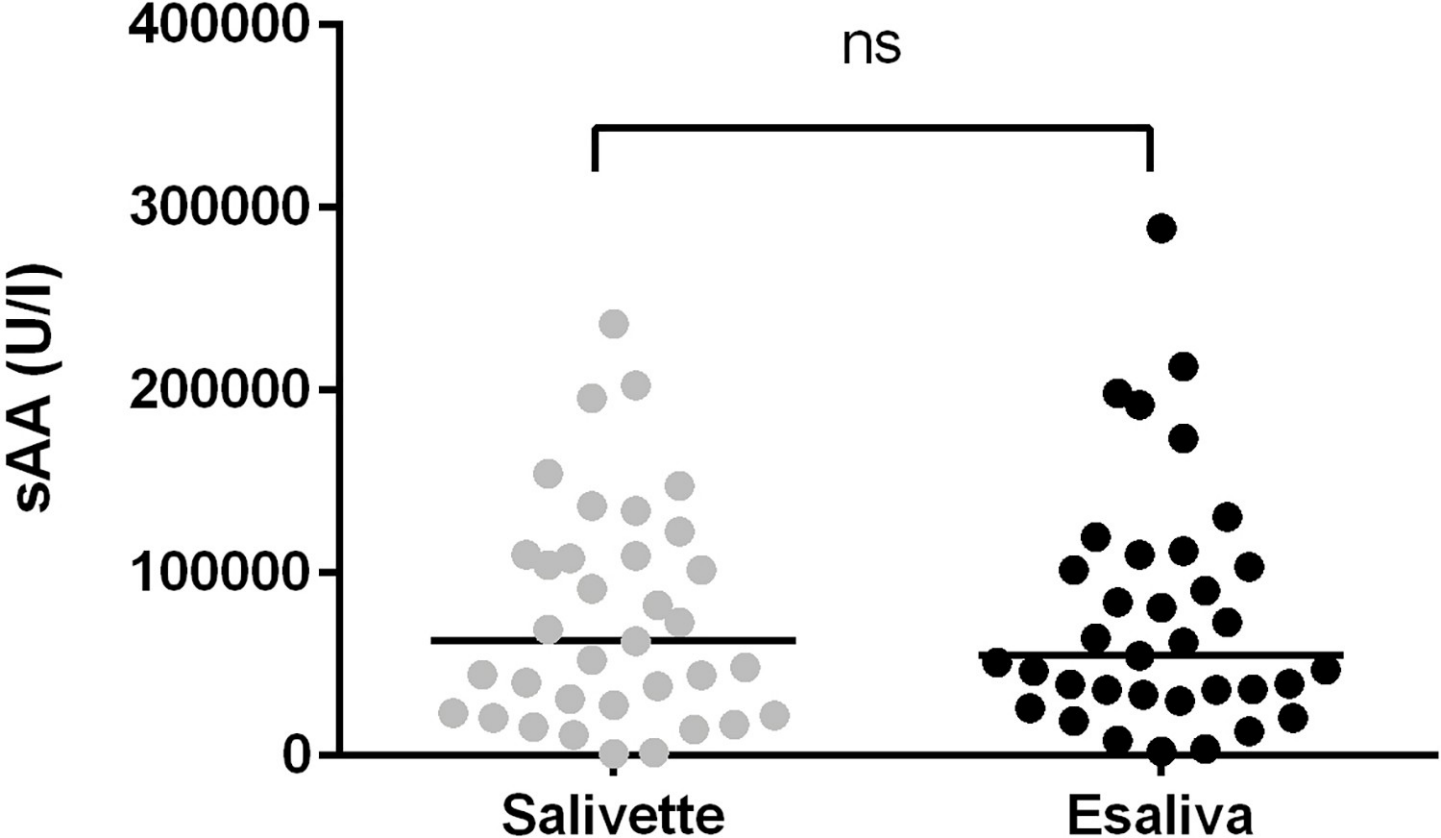
Comparison of the levels of amylase activity measured in saliva samples collected by 35 residents with Salivette and Esaliva devices. The dots represent data distribution, black line represents median sAA levels.

No significant differences were observed in the levels of sAA between Salivette and Esaliva (W = 18.00, p = 0.8826) (Fig 3).

Based on these results and to avoid redundancy, only the results obtained from sample collected with the Salivette device will be presented.

During a typical working day, all residents collected two saliva samples 10 minutes apart, as the collection scheme used before and after the simulation. No significant difference between sAA levels were observed (W = -160.0, p = 0.0698) (S2 Fig).

### sAA as biomarker of stress

**Fig 4:**
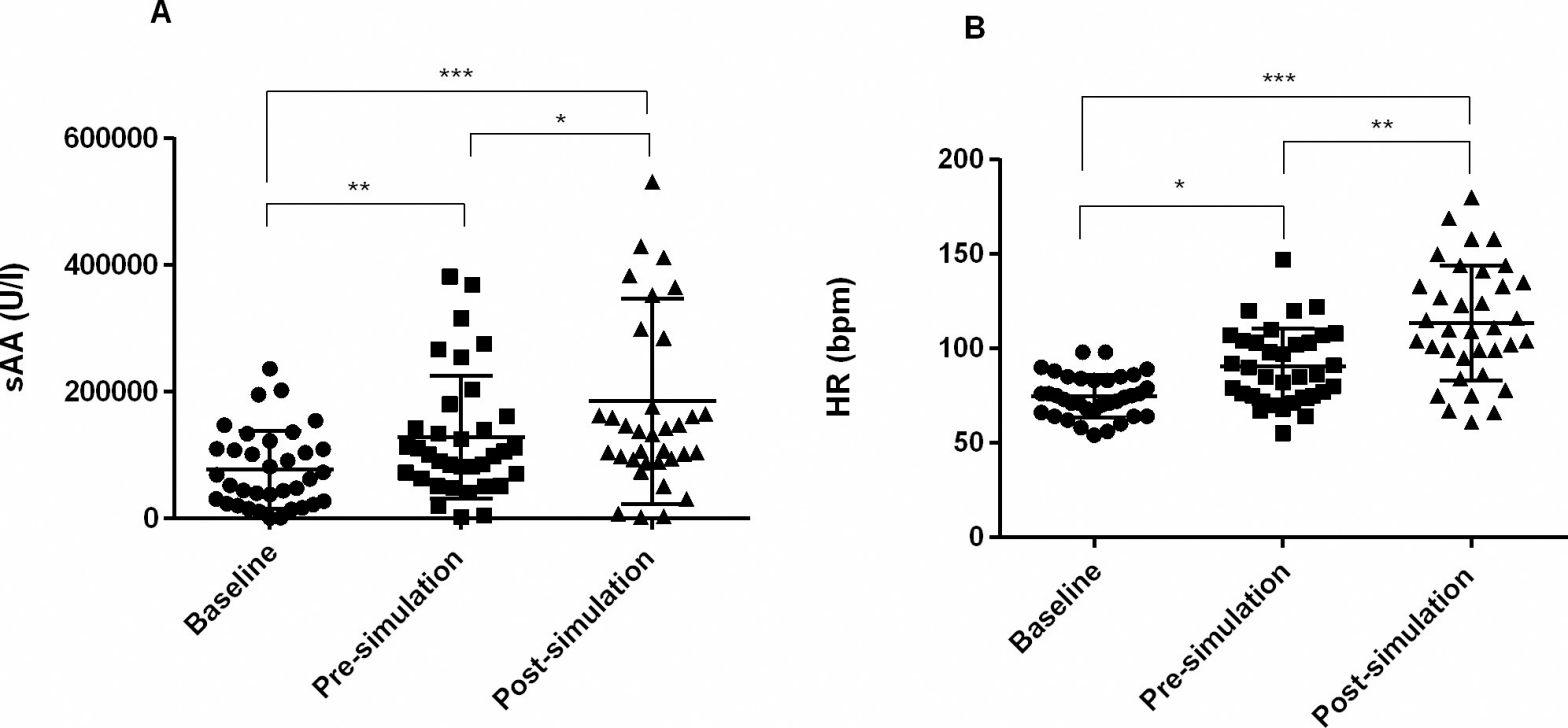
sAA and heart rate response to stress on simulation day. sAA activity (A) and heart rate (B) measured in gynecologist residents at baseline (8.00 am), before and after the simulation of traumatic birth scenario. sAA results were obtained by the AMYL2 analytical method. Significant differences are represented as *p<0.05; **p<0.005; ***p<0.0005. bpm = beats per minute.

To determine whether sAA can serve as a stress biomarker, 35 gynecologist residents were tested on a day when they were scheduled to manage one of the most traumatic birth events in their professional lives: a simulated shoulder dystocia (simulation day). Saliva samples were obtained at 8.00 am (baseline), immediately before and after the simulation. Fig 4A shows that sAA activity levels measured after the simulation were significantly higher than those measured before the scenario took place, about ten minutes later (F = 41.83, p<0.0001). A similar behavior was observed when HR was considered (F = 46.46, p<0.0001) (Fig 4B).

**Fig 5:**
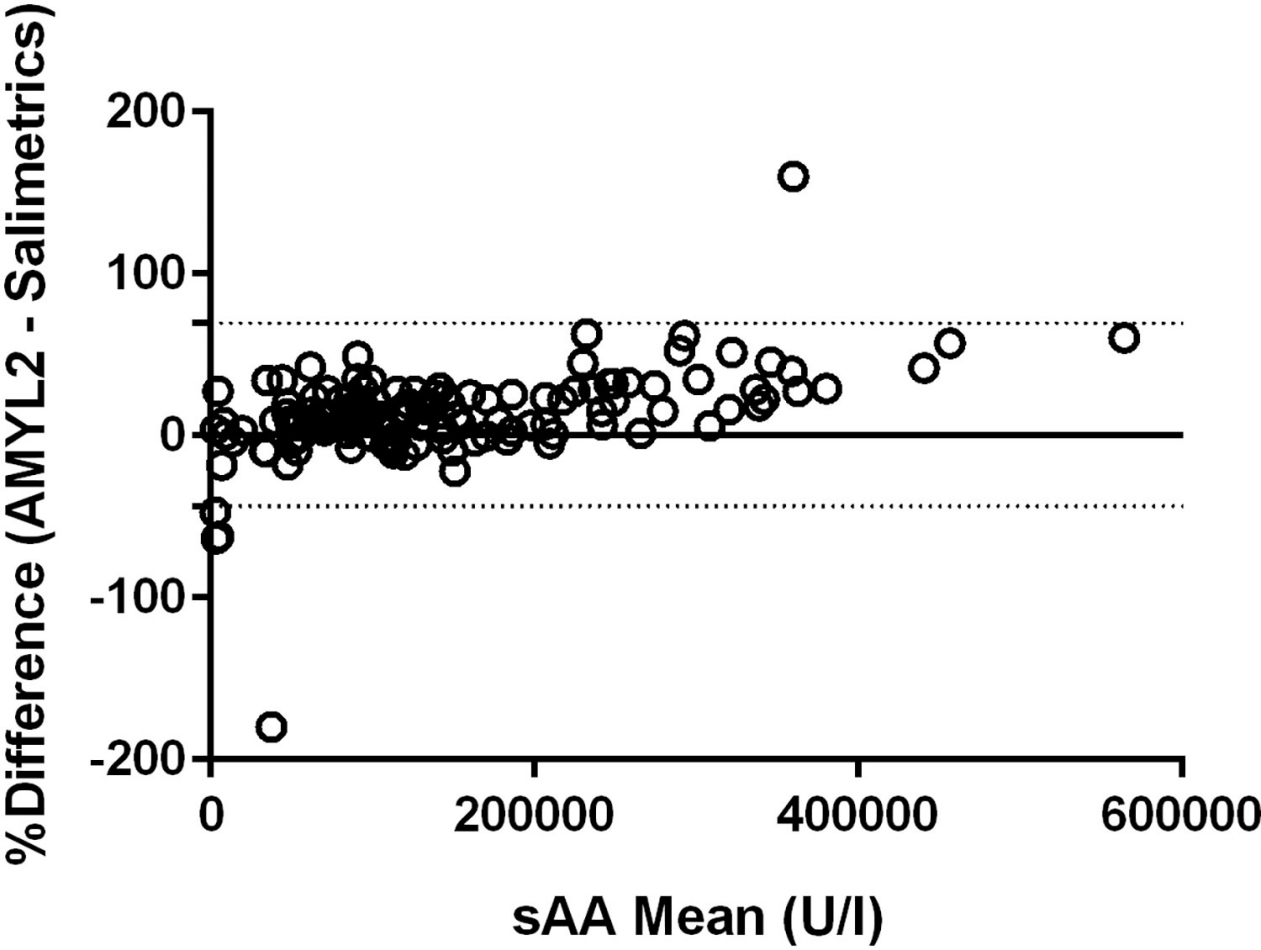
Bland and Altman plot of differences between the AMYL2 assay compared to Salimetrics assay for the analysis of sAA, expressed as percentages of the values on the axis [(AMYL2 –Salimetrics/mean%)], vs. the mean of the two measurements. The mean was represented by the black line. The Upper and Lower Bland Altman 95% Limit of agreement were represented by the grey dotted line.

The two assays AMYL2 and Salimetrics, were compared using the residents’ saliva samples obtained on simulation day before and after the simulation scenario, and those paired samples obtained in a different working day. A highly significant correlation exists between the two different assays measuring sAA activity (r = 0.954; p = 0.000). Bland Atman analysis showed a bias (mean difference) of 12.80%, almost constant for all the measured concentrations, except for high values. The agreement limits ranged from -43.72 to 69.32 (Fig 5).

## Discussion

sAA has been recently investigated as a biomarker of stress as an alternative to cortisol, due to its quicker activity level variation in response to stressful stimuli, reflecting the SAM activity. It is produced locally in the salivary glands, and its secretion, like other secreted components of whole saliva, could be influenced by the circadian rhythm and the state of stimulation of the salivary glands [15, 21].

In this context, we verified intra and inter-day variations of sAA activity levels before proposing sAA as a biomarker of stress. Our findings suggest a typical diurnal pattern of sAA levels, with significant increases from morning to afternoon, which is consistent with earlier studies [19, 24–27]. In contrast, no significant inter-day variation was observed in the sAA levels, as also confirmed by Kawanishi et al. [28] in a study on salivary metabolomic profiles. Therefore, it is suggested that sampling time should be tightly controlled to minimize unexpected bias in the clinical use of not only sAA but also other salivary metabolites.

In addition to diurnal changes, we also observed significant inter-individual differences in sAA levels. These differences may be attributed to individual traits and the ability to respond emotionally to stressful situations based on the individual temperament, as suggested by Doane and Van Lenten’s study of sAA variations in adolescents [27]. Quintana et al. also found inter-individual variability in salivary protein patterns [29]. This variability included enzymes involved in carbohydrate metabolism such as amylase and glycolytic enzymes, as well as other proteins that participate in the non-specific and specific immune response. These results also suggest that inter-individual variability should be accounted for when searching for salivary biomarkers or studying in-mouth biochemical mechanisms.

Gender effects on sAA level are contrastive [26, 30, 31]. According to Nater et al. [19], we observed no differences in sAA basal levels between males and females. The discrepant results documented in literature might be due to several factors as the time of day, menstrual cycle phase in women, and lifestyle habits (e.g. physical exercise, medical drugs and caffeine consumption, smoking), and the number of studied participants, so universal conclusions cannot be made.

In addition, the composition of saliva can be affected by various factors such as collection procedures (including unstimulated and stimulated methods), collection devices, oral hygiene, and food [32, 33]. To minimize methodological bias and investigate new collection devices, this study evaluated two different devices: Salivette and Esaliva. Salivette is a widely used device which might become hazardous for children and in some context of the elderly individuals who may accidentally swallow the swab. To overcome this limitation, we evaluated a new commercially available device called Esaliva, which is equipped with a rod to which a swab is attached.

No significant difference in sAA activity level was observed when comparing Salivette and Esaliva. This is likely due to the fact that saliva samples were collected using both devices after a mechanical stimulation procedure, and then centrifuged under the same conditions of time and speed. However, some residents were unable to collect all the requested samples using the Esaliva device. This phenomenon might depend on differences in salivary flow rate and on the saliva collection method [34]. Differences in salivary flow rate and composition are well known and they have been related not only to stressful situation compared to the real life, but also to the sources and intensity of stress [35]. sAA normalization with respect to flow rate or protein concentration have been suggested [36] and Contreras-Aguilar et al. [37] demonstrated that the way of reporting sAA can influence the results obtained in different stress models and also their interpretation. However, another study [38] comparing activity and flow rate did not find differences in stress responses between both measures.

Our findings suggest that, in addition to circadian rhythm, it is crucial to evaluate the collection method and device properly and standardize this process before using sAA in clinical evaluations.

Based on these preliminary results, we aimed to ascertain the role of sAA as biomarker of stress.

To achieve this, we enrolled 35 gynecologist residents and simulated a stressful working scenario, namely shoulder dystocia. This is defined as a vaginal cephalic delivery that requires additional obstetric manoeuvres following failure to deliver the shoulders after gentle downward traction on the bay’s head [39]. Since shoulder dystocia causes considerable neonatal and maternal morbidity, and even death of the newborn [40], it is considered one of the most traumatic birth experiences not only for women, but also for clinicians involved [41].

From the perspective of midwives and clinicians, the involvement in birth trauma may have long term negative impact. Fear, anxiety, guilt, shame, vulnerability and reduced professional confidence are among the most common feelings experienced by midwives after a traumatic birth [42, 43] Adverse outcomes may go beyond these, and include post-trauma stress disorder, compassion fatigue, burnout, and workforce attrition [44]. To date, the level of stress experienced by the clinicians and midwives during shoulder dystocia was never assessed. Thus, we collected saliva samples from residents experiencing the simulated shoulder dystocia scenario at specific time points to measure stress levels: a) at 8.00 a.m., b) before the simulation scenario and c) after the simulation scenario (approximately ten minutes later). Notably, pre-simulation sAA levels were significantly higher than baseline values and lower than sAA levels measured only ten minutes after the simulation scenario. This pattern mirrored heart rate this supporting sAA as biomarker of stress. Moreover, our data support the hypothesis that stress started before the simulation and it was magnified during the simulation. On the contrary, when residents collected saliva repeatedly during a normal day without any stressful stimuli, no variations were observed at the same collection time points. Peek et al., in neonatal medicine trainees, also observed that simulated events were associated with greater likelihood of threat appraisal and higher state anxiety with respect to real world emergencies. Possible explanations include participants’ previous experiences and expectations of simulation and the effect of post-simulation feedback [45].

All these results were confirmed by two different methods, suggesting that sAA represents not only a reliable marker of response to stress stimuli but also a rapid responder marker making it an ideal marker for capturing the intense stress experienced during such critical events. Engert et al [46] observed similar results, showing that sAA levels in adults peak 10 minutes after psychological stress and return to baseline at around 50 minutes and Angeli et al [25] also observed an increase in mean sAA levels ten minutes after venipuncture in children. This specificity and reliability underscore the potential of sAA as a tool not only for identify acute stress sources but also for guiding interventions aimed at reducing the adverse effects of acute stress in obstetric care, as in other context.

Finally, to any biomarker to be used in a clinical context, it is important that it can be measured easily, accurately, and quickly, with results promptly available. In the light of these considerations, we evaluated two different analytical methods: AMYL2 and Salimetrics. The first method, AMYL2, is an enzymatic colorimetric assay for the quantitative determination of α-amylase activity, that is performed in total automation using the Roche Cobas 8000 analyzer. This method is validated by the manufacturer for use with human serum, plasma, and urine matrices, but not for saliva samples. However, our evaluation of analytical performance, reported as supporting information, supports the use of the AMYL2 assay, with prior sample dilution, to determine sAA activity. As sAA is the main protein in saliva (40–50% of total proteins) [47–49], it is necessary to dilute saliva samples before applying kinetic colorimetric tests to measure sAA within the analytical range of the assay. In this study, we choose to dilute all saliva samples 1:100 after test linearity under dilution, by using the manufacturer-recommended sample diluent (NaCl 9%) to rerun samples with high activity. Although sample dilution can introduce measurement inaccuracy, our results, suggest a very good precision (CV equal to 7%), comparable to that reported by other Authors [50, 51]. We also found that only highly diluted saliva samples (1:800 and 1:1600) resulted in reduced sAA activity. These findings are consistent with those reported by Skoluda et al [52] who demonstrated that the instability of sAA activity in diluted samples is determined by the interaction of material, diluent and time. They proved that sAA activity remains stable when NaCl or PBS are used as diluent. On the contrary, they observed a time-dependent decline when ultra-pure water was used as a diluent that could be avoided by processing samples immediately after dilution (within 25 minutes). Moreover, our results are consistent with those obtained by Angeli et al [25] using the same collection method (Salivette) and the same assay. The second method, Salimetrics, is a kinetic enzyme assay kit optimized for use with saliva sample, but is not automated. There was a high correlation between the two methods, and both could be used to measure sAA activity in saliva samples. However, AMYL2 has several advantages over the Salimetric assay for a fast determination of sAA: a) hundreds of measurements within a time period ranging from minutes to hours; b) complete automation of the analysis, including the pre-dilution of the sample and safe handling procedures. Recently, a new method able to measure specific proteoforms which might reflect different stressful situation has been proposed, and it should be taken in consideration for future studies [53].

The present study has some potential limitations. Firstly, we did not analyse other salivary markers of stress, such as cortisol, in comparison to sAA. However, due to the small amount of collected saliva, we decided to use the samples to determine which collection device or analytical method would be preferable for sAA determination. However, stress levels were also evaluated through the HR measurement. Finally, we could not measure salivary flow rate, which may influence sAA. However, our findings are assumed to be somewhat acceptable since much variation was not found in the saliva flow rates of similarly aged populations [54].

## Conclusions

In conclusion, salivary alpha-amylase (sAA) activity represents an accurate and rapid marker for measuring response to stress in different clinical settings, including psychology and neuroscience. This study also highlights the importance of carefully evaluating the sample collection time before introducing sAA in a clinical setting. Finally, the results indicate the feasibility of a saliva-based approach to detect new biomarkers, contributing to the advancement of healthcare and diagnosis.

## Supporting information

S1 File Materials, methods and results on the analytical performances of the assay in saliva samples. (PDF)

S1 Fig Linearity under dilution of two saliva samples with two different sAA activity levels. (TIF)

S2 Fig sAA levels in two saliva samples collected by residents after ten minutes each other. sAA results obtained by AMYL2 methods (ns = no significant statistically differences were found).
(TIF)

S1 Table Linearity of dilution. The Table shows the measured sAA activity levels of two saliva samples serially diluted versus the expected levels at certain dilutions.
(PDF)

## References

[1] Cañete MG, Valenzuela IM, Garcés PC, Massó IC, González MJ, Providell SG. Saliva sample for the massive screening of SARS-CoV-2 infection: a systematic review. Oral Surg Oral Med Oral Pathol Oral Radiol. 2021;131:540–548. doi: 10.1016/j.oooo.2021.01.028 33637473 PMC7849433

[2] Dongiovanni P, Meroni M, Casati S, Goldoni R, Thomaz DV, Kehr NS, et al. Salivary biomarkers: novel noninvasive tools to diagnose chronic inflammation. Int J Oral Sci. 2023;15:27. doi: 10.1038/s41368-023-00231-6 37386003 PMC10310701

[3] Yoshizawa JM, Schafer CA, Schafer JJ, Farrell JJ, Paster BJ, Wong DT. Salivary biomarkers: toward future clinical and diagnostic utilities. Clin Microbiol Rev. 2013;26:781–91. doi: 10.1128/CMR.00021-13 24092855 PMC3811231

[4] Kaufman E, Lamster IB. The diagnostic applications of saliva—a review. Crit Rev Oral Biol Med. 2002;13:197–212. doi: 10.1177/154411130201300209 12097361

[5] Chojnowska S, Ptaszyńska-Sarosiek I, Kępka A, Knaś M, Waszkiewicz N. Salivary Biomarkers of Stress, Anxiety and Depression. J Clin Med. 2021;10:517. doi: 10.3390/jcm10030517 33535653 PMC7867141

[6] Hellhammer DH, Wüst S, Kudielka BM. Salivary cortisol as a biomarker in stress research. Psychoneuroendocrinology. 2009;34:163–171. doi: 10.1016/j.psyneuen.2008.10.026 19095358

[7] van Stegeren A, Rohleder N, Everaerd W, Wolf OT. Salivary alpha amylase as marker for adrenergic activity during stress: effect of betablockade. Psychoneuroendocrinology. 2006;31:137–41. doi: 10.1016/j.psyneuen.2005.05.012 16046076

[8] Herman JP, McKlveen JM, Ghosal S, Kopp B, Wulsin A, Makinson R, et al. Regulation of the Hypothalamic-Pituitary-Adrenocortical Stress Response. Compr Physiol. 2016;6:603–21. doi: 10.1002/cphy.c150015 27065163 PMC4867107

[9] Armario A, Labad J, Nadal R. Focusing attention on biological markers of acute stressor intensity: Empirical evidence and limitations. Neurosci Biobehav Rev. 2020;111:95–103. doi: 10.1016/j.neubiorev.2020.01.013 31954151

[10] Chrousos GP, Gold PW. The concepts of stress and stress system disorders. Overview of physical and behavioral homeostasis. JAMA. 1992;267:1244–52. Erratum in: JAMA 1992;268:200. 1538563

[11] Bañuelos MS, Musleh A, Olson LE. Measuring Salivary Alpha-Amylase in the Undergraduate Neuroscience Laboratory. J Undergrad Neurosci Educ. 2017;16:A23–A27. 29371837 PMC5777833

[12] Kanikowska D, Roszak M, Rutkowski R, Sato M, Sikorska D, Orzechowska Z, et al. Seasonal differences in rhythmicity of salivary cortisol in healthy adults. J Appl Physiol (1985). 2019;126:764–770. doi: 10.1152/japplphysiol.00972.2018 30702977

[13] Casals G, Foj L, de Osaba MJ. Day-to-day variation of late-night salivary cortisol in healthy voluntaries. Clin Biochem. 2011;44:665–8. doi: 10.1016/j.clinbiochem.2011.02.003 21320479

[14] Chatterton RT Jr, Vogelsong KM, Lu YC, Ellman AB, Hudgens GA. Salivary alpha-amylase as a measure of endogenous adrenergic activity. Clin Physiol. 1996;16:433–48. doi: 10.1111/j.1475-097x.1996.tb00731.x 8842578

[15] Nater UM, Rohleder N. Salivary alpha-amylase as a non-invasive biomarker for the sympathetic nervous system: current state of research. Psychoneuroendocrinology. 2009;34:486–96. doi: 10.1016/j.psyneuen.2009.01.014 19249160

[16] Bhattarai Kashi Raj, Junjappa Raghupatil, Handigund Mallikarjun, Kim Hyung-Ryong, Chae Han-Jung. Alpha Amylase Saliva Isoenzyme—an overview. Autoimmunity Reviews. 2022; 17; 376–390. 10.1016/j.autrev.2017.11.031 29428807

[17] O’Donnell MD, Miller NJ. Plasma pancreatic and salivary-type amylase and immunoreactive trypsin concentrations: variations with age and reference ranges for children. Clin Chim Acta. 1980;104:265–73. doi: 10.1016/0009-8981(80)90384-8 6156037

[18] Nater UM, Hoppmann CA, Scott SB. Diurnal profiles of salivary cortisol and alpha-amylase change across the adult lifespan: evidence from repeated daily life assessments. Psychoneuroendocrinology. 2013;38:3167–71. doi: 10.1016/j.psyneuen.2013.09.008 24099860 PMC3844069

[19] Nater UM, Rohleder N, Schlotz W, Ehlert U, Kirschbaum C. Determinants of the diurnal course of salivary alpha-amylase. Psychoneuroendocrinology. 2007;32:392–401. doi: 10.1016/j.psyneuen.2007.02.007 17418498

[20] Jantaratnotai N, Rungnapapaisarn K, Ratanachamnong P, Pachimsawat P. Comparison of salivary cortisol, amylase, and chromogranin A diurnal profiles in healthy volunteers. Arch Oral Biol. 2022;142:105516. doi: 10.1016/j.archoralbio.2022.105516 35952574

[21] Rohleder N, Nater UM. Determinants of salivary alpha-amylase in humans and methodological considerations. Psychoneuroendocrinology. 2009;34:469–85. doi: 10.1016/j.psyneuen.2008.12.004 19155141

[22] Ameringer S, Munro C, Elswick RK Jr. Assessing agreement between salivary alpha amylase levels collected by passive drool and eluted filter paper in adolescents with cancer. Oncol Nurs Forum. 2012;39:E317–23. doi: 10.1188/12.ONF.E317-E323 22750901 PMC4049331

[23] Takagi K, Ishikura Y, Hiramatsu M, Nakamura K, Degawa M. Development of a saliva collection device for use in the field. Clin Chim Acta. 2013;425:181–5. doi: 10.1016/j.cca.2013.08.008 23954838

[24] Xie H, Zheng X, Huang Y, Li W, Wang W, Li Q, et al. Diurnal pattern of salivary alpha-amylase and cortisol under citric acid stimulation in young adults. PeerJ. 2022;10:e13178. doi: 10.7717/peerj.13178 35433126 PMC9012170

[25] Angeli E, Korpa T, Johnson EO, Apostolakou F, Papassotiriou I, Chrousos GP, et al. Salivary cortisol and alpha-amylase diurnal profiles and stress reactivity in children with Attention Deficit Hyperactivity Disorder. Psychoneuroendocrinology. 2018;90:174–181. doi: 10.1016/j.psyneuen.2018.02.026 29501948

[26] Pérez-Valdecantos D, Caballero-García A, Del Castillo-Sanz T, Bello HJ, Roche E, Córdova A. Stress Salivary Biomarkers Variation during the Work Day in Emergencies in Healthcare Professionals. Int J Environ Res Public Health. 2021;18:3937. doi: 10.3390/ijerph18083937 33918537 PMC8070075

[27] Doane LD, Van Lenten SA. Multiple time courses of salivary alpha-amylase and dimensions of affect in adolescence. Psychoneuroendocrinology. 2014;49:47–53. doi: 10.1016/j.psyneuen.2014.06.007 25076484

[28] Kawanishi N, Hoshi N, Masahiro S, Enomoto A, Ota S, Kaneko M, et al. Effects of inter-day and intra-day variation on salivary metabolomic profiles. Clin Chim Acta. 2019;489:41–48. doi: 10.1016/j.cca.2018.11.030 30481500

[29] Quintana M, Palicki O, Lucchi G, Ducoroy P, Chambon C, Salles C, et al. Inter-individual variability of protein patterns in saliva of healthy adults. J Proteomics. 2009;72:822–30. doi: 10.1016/j.jprot.2009.05.004 19470415

[30] Carr AR, Scully A, Webb M, Felmingham KL. Gender differences in salivary alpha-amylase and attentional bias towards negative facial expressions following acute stress induction. Cogn Emot. 2016;30:315–24. doi: 10.1080/02699931.2014.999748 25787848

[31] Rutherfurd-Markwick K, Starck C, Dulson DK, Ali A. Salivary diagnostic markers in males and females during rest and exercise. J Int Soc Sports Nutr 2017;14: 27. doi: 10.1186/s12970-017-0185-8 28811748 PMC5553796

[32] Al Habobe H, Haverkort EB, Nazmi K, Van Splunter AP, Pieters RHH, Bikker FJ. The impact of saliva collection methods on measured salivary biomarker levels. Clin Chim Acta. 2024;552:117628. doi: 10.1016/j.cca.2023.117628 37931731

[33] Helmerhorst EJ, Dawes C, Oppenheim FG. The complexity of oral physiology and its impact on salivary diagnostics. Oral Dis. 2018;24:363–371. doi: 10.1111/odi.12780 28922514 PMC6858792

[34] Topkas E, Keith P, Dimeski G, Cooper-White J, Punyadeera C. Evaluation of saliva collection devices for the analysis of proteins. Clin Chim Acta. 2012;413:1066–70. doi: 10.1016/j.cca.2012.02.020 22405932

[35] Matos-Gomes N, Katsurayama M, Makimoto FH, Santana LL, Paredes-Garcia E, Becker MA, et al. Psychological stress and its influence on salivary flow rate, total protein concentration and IgA, IgG and IgM titers. Neuroimmunomodulation. 2010;17:396–404. doi: 10.1159/000292064 20516721

[36] Strahler J, Skoluda N, Kappert MB, Nater UM. Simultaneous measurement of salivary cortisol and alpha-amylase: Application and recommendations. Neurosci Biobehav Rev. 2017;83:657–677. doi: 10.1016/j.neubiorev.2017.08.015 28864234

[37] Contreras-Aguilar MD, Escribano D, Martínez-Subiela S, Martínez-Miró S, Rubio M, Tvarijonaviciute A, et al. Influence of the way of reporting alpha-Amylase values in saliva in different naturalistic situations: A pilot study. PLoS One. 2017;12:e0180100. doi: 10.1371/journal.pone.0180100 28654668 PMC5487069

[38] Rohleder N, Wolf JM, Maldonado EF, Kirschbaum C. The psychosocial stress-induced increase in salivary alpha-amylase is independent of saliva flow rate. Psychophysiology. 2006;43:645–52. doi: 10.1111/j.1469-8986.2006.00457.x 17076822

[39] Resnik R. Management of shoulder girdle dystocia. Clin Obstet Gynecol 1980;23:559–64. doi: 10.1097/00003081-198006000-00024 6994971

[40] Dajani NK, Magann EF. Complications of shoulder dystocia. Semin Perinatol 2014;38:201–4. doi: 10.1053/j.semperi.2014.04.005 24863025

[41] Minooee S, Cummins A, Foureur M, Travaglia J. Shoulder dystocia: A panic station or an opportunity for post-traumatic growth? Midwifery 2021;101:103044. doi: 10.1016/j.midw.2021.103044 34098223

[42] Sheen K, Spiby H, Slade P. The experience and impact of traumatic perinatal event experiences in midwives: A qualitative investigation. Int J Nurs Stud 2016;53:61–72. doi: 10.1016/j.ijnurstu.2015.10.003 26546399

[43] Wahlberg Å, Högberg U, Emmelin M. The erratic pathway to regaining a professional self-image after an obstetric work-related trauma: A grounded theory study. Int J Nurs Stud 2019;89:53–61. doi: 10.1016/j.ijnurstu.2018.07.016 30342325

[44] Leinweber J, Creedy DK, Rowe H, Gamble J. A socioecological model of posttraumatic stress among Australian midwives. Midwifery 2017;45:7–13. doi: 10.1016/j.midw.2016.12.001 27960122

[45] Peek R, Moore L, Arnold R. Psychophysiological fidelity: A comparative study of stress responses to real and simulated clinical emergencies. Med Educ 2023;57:1248–1256. doi: 10.1111/medu.15155 37392166 PMC10946833

[46] Engert V, Vogel S, Efanov SI, Duchesne A, Corbo V, Ali N, et al. Investigation into the cross-correlation of salivary cortisol and alpha-amylase responses to psychological stress. Psychoneuroendocrinology. 2011;36:1294–302. doi: 10.1016/j.psyneuen.2011.02.018 21470780

[47] Oppenheim FG, Salih E, Siqueira WL, Zhang W, Helmerhorst EJ. Salivary proteome and its genetic polymorphisms. Ann N Y Acad Sci. 2007;1098:22–50. doi: 10.1196/annals.1384.030 17303824

[48] Humphrey SP, Williamson RT. A review of saliva: normal composition, flow, and function. J Prosthet Dent. 2001;85:162–9. doi: 10.1067/mpr.2001.113778 11208206

[49] Noble RE. Salivary alpha-amylase and lysozyme levels: a non-invasive technique for measuring parotid vs submandibular/sublingual gland activity. J Oral Sci. 2000;42:83–6. doi: 10.2334/josnusd.42.83 10989590

[50] Fuentes M, Tecles F, Gutiérrez A, Otal J, Martínez-Subiela S, Cerón JJ. Validation of an automated method for salivary alpha-amylase measurements in pigs (Sus scrofa domesticus) and its application as a stress biomarker. J Vet Diagn Invest. 2011;23:282–7. doi: 10.1177/104063871102300213 21398448

[51] Fuentes-Rubio M, Fuentes F, Otal J, Quiles A, Hevia ML. Validation of an assay for quantification of alpha-amylase in saliva of sheep. Can J Vet Res. 2016;80:197–202. 27408332 PMC4924553

[52] Skoluda N, Dhrami I, Nater UM. Factors contributing to stability and instability in alpha-amylase activity in diluted saliva samples over time. Psychoneuroendocrinology. 2020;121:104847. doi: 10.1016/j.psyneuen.2020.104847 32889490

[53] Contreras-Aguilar MD, Mateo SV, Tecles F, Hirtz C, Escribano D, Cerón JJ. Changes Occurring on the Activity of Salivary Alpha-Amylase Proteoforms in Two Naturalistic Situations Using a Spectrophotometric Assay. Biology (Basel). 2021;10:227. doi: 10.3390/biology10030227 33809418 PMC7999747

[54] Sahu GK, Upadhyay S, Panna SM. salivary alpha amylase activity in human beings of different age groups subjected to psychological stress. Indian J Clin Biochem. 2014;29:485–490. doi: 10.1007/s12291-013-0388-y 25298630 PMC4175699

