# Supplementary material for: Salivary alpha-amylase: A marker of stress in gynecological residents during a shoulder dystocia simulation scenario
Source: PLoS One. 2024 Nov 25;19(11):e0314234. doi: 10.1371/journal.pone.0314234 (PMC11588261; doi:10.1371/journal.pone.0314234)
Supplement: S1 File — (PDF) [file pone.0314234.s001.pdf]

S1 File

Materials and Methods

Data on the evaluation of analytical performances of the assay in saliva samples, focused on the evaluation of the dilution used, were also obtained. The linearity of dilution was evaluated by serially diluting 2 human salivary samples (1:50, 1:100, 1:200, 1:400, 1:800, 1:1600). The measured sAA activity levels were compared with the expected levels.

Recovery evaluation was carried out using two human saliva specimens in duplicate. One sample with low sAA activity level was diluted 1:200 in NaCl 9%, to reach a lower-level activity, and split into 12 sample aliquots. A second sample with high sAA activity level was added in increasing amount (5 µl, 10 µl, 15 µl, 20 µl, 25 µl, 30 µl) to the 6 different aliquots of sample at low sAA activity to achieve saliva concentration increments. The final sAA activity levels were re-estimated, taking into account the added quantities and the final volumes.

The repeatability on diluted samples was also assessed. Within-run imprecision was defined as the coefficient of variation (CV) of 5 replicate determinations of 2 human saliva samples diluted 1:100 in one run. Between-run imprecision was also defined by the CV measured with 5 replicate determinations of the same 2 saliva samples in 3 separate days. Regression analysis was used to analyze linearity.

Results

Alpha-amylase activity levels: assay performance in saliva

For dilution analysis, two different saliva samples were serially diluted with the sample diluent provided by the manufacturer (NaCl 9%) to test linearity. The correlation coefficient was 0.9988 and 0.9991, respectively, as shown in S1 Fig. The percentage yield was greater than 90% for dilutions

1:100 and 1:200, and greater than 75% for dilution 1:400 (S1 Table). In a separate experiment, two saliva samples were mixed in varying proportions by adding small volumes of one sample, which showed high sAA activity levels, to the other sample with low sAA activity levels. This was done to evaluate recovery. The average recovery value was 100±3% (n=12). The within-run coefficients of variation calculated for two saliva samples diluted 1:100 (sample 1: 1151.2±80.1 U/L; sample 2: 1251.8±81.9 U/L) were 7% and 6.5% respectively. The between-run coefficients of variation of two saliva samples (sample 1: 1168.5±81.3 U/L; sample 2: 1275.9±83.5 U/L) were also 7% and 6.5% respectively.
