# Supplementary figures and images for: Salivary alpha-amylase: A marker of stress in gynecological residents during a shoulder dystocia simulation scenario
Source: PLoS One. 2024 Nov 25;19(11):e0314234. doi: 10.1371/journal.pone.0314234 (PMC11588261; doi:10.1371/journal.pone.0314234)

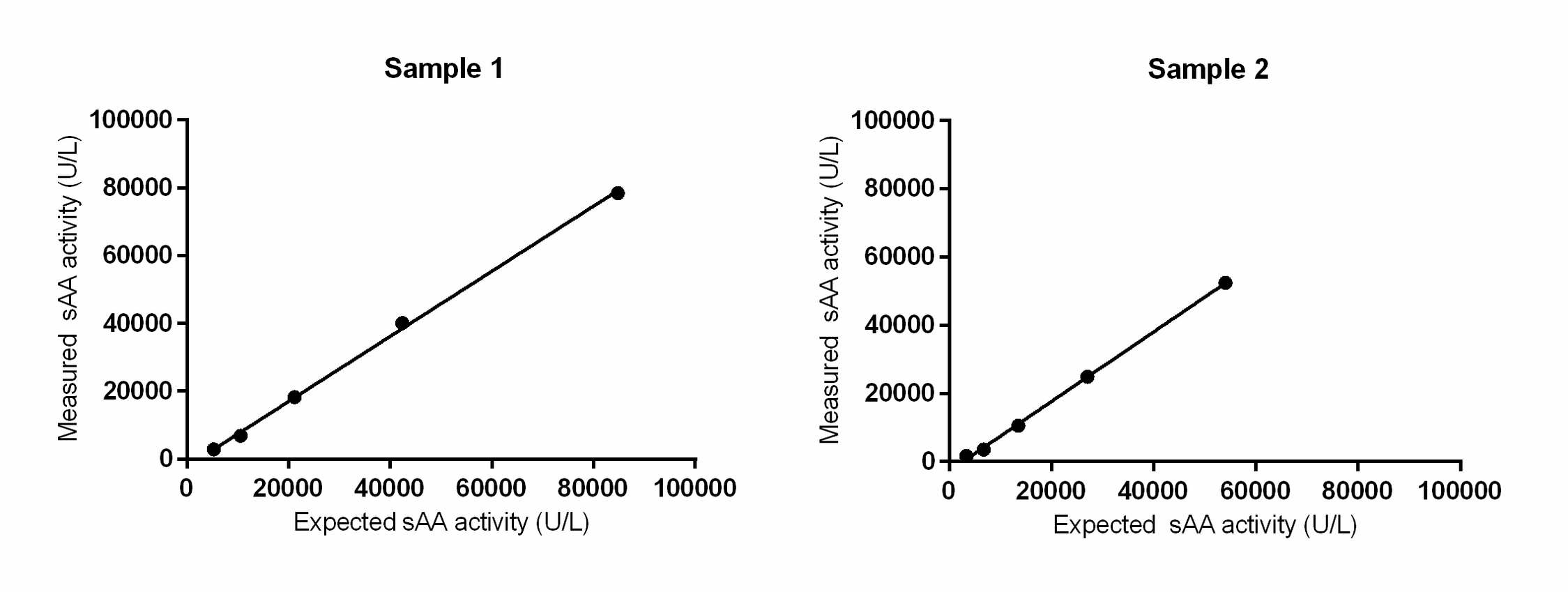

Supplement: S1 Fig — (TIF) [file pone.0314234.s002.tif]

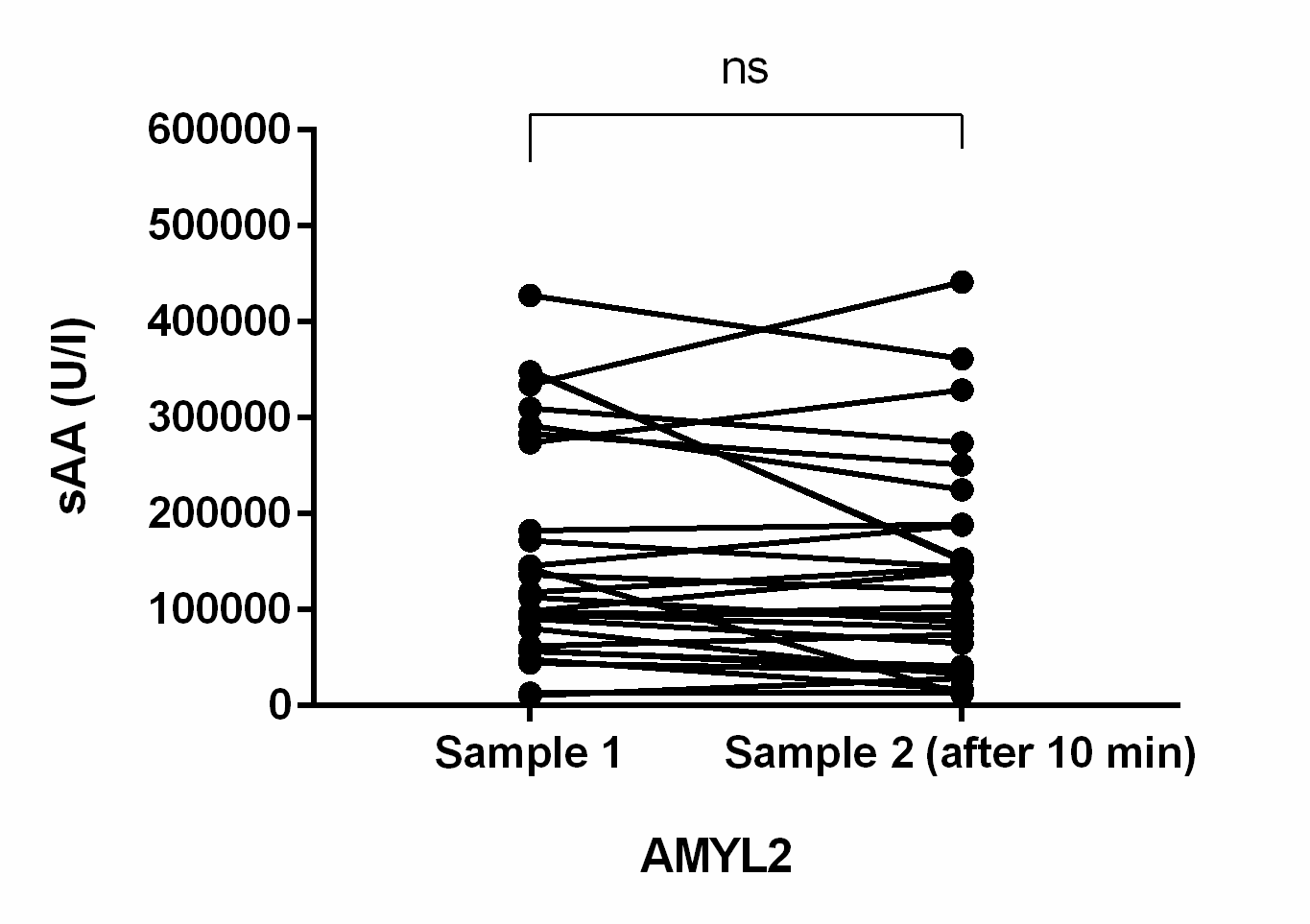

Supplement: S2 Fig — sAA results obtained by AMYL2 methods (ns = no significant statistically differences were found). (TIF) [file pone.0314234.s003.tif]
