# Supplementary material for: Salivary alpha-amylase: A marker of stress in gynecological residents during a shoulder dystocia simulation scenario
Source: PLoS One. 2024 Nov 25;19(11):e0314234. doi: 10.1371/journal.pone.0314234 (PMC11588261; doi:10.1371/journal.pone.0314234)
Supplement: S1 Table — The Table shows the measured sAA activity levels of two saliva samples serially diluted versus the expected levels at certain dilutions. (PDF) [file pone.0314234.s004.pdf]

S1 Table. Linearity of dilution. The table shows the measured sAA activity levels of two saliva samples serially diluted versus the expected levels at certain dilutions.

	**Dilution**	**Measured value (U/L)**	**Expected value (U/L)**	**Percent yield**
**Saliva 1**				
	1:50	3389	/	
	1:100	1569	1695	93%
	1:200	803	847	95%
	1:400	366	424	86%
	1:800	137	212	65%
	1:1600	57	106	54%
**Saliva 2**				
	1:50	2161	/	
	1:100	1049	1081	97%
	1:200	498	540	92%
	1:400	210	270	78%
	1:800	70	135	52%
	1:1600	33	68	49%
